# Oral risedronate increases Gruen zone bone mineral density after primary total hip arthroplasty: a meta-analysis

**DOI:** 10.1186/s13018-018-0794-1

**Published:** 2018-06-07

**Authors:** Qifeng Li, Baoshan Xu

**Affiliations:** 1 Graduate school, Tianjin Medical University, No. 22, Qixiangtai road, Heping district, Tianjin, China; 2 Department of orthopaedics, Tianjin Hospital, 406. No, Jiefangnan Road, Hexi district, Tianjin, 300000 China

**Keywords:** Risedronate, Total hip arthroplasty, Meta-analysis

## Abstract

**Background:**

This meta-analysis was performed to assess the efficacy of risedronate in increasing bone mineral density (BMD) in patients undergoing primary total hip arthroplasty (THA).

**Methods:**

We systematically searched the following databases: PubMed, Embase, Web of Science, Cochrane Library, and Chinese Wanfang database from inception up to October 2017. Included patients were prepared for THA and were separated into two groups: intervention group (risedronate treatment) and control group (placebo treatment). BMD change in Gruen zone 1 and 7 were primary outcomes. Meta-analysis was performed using Stata 12.0 software.

**Results:**

Five randomized controlled trials (RCTs) involving 259 patients (risedronate group = 127, control group = 132) were finally included in this meta-analysis. Meta-analysis indicated that oral risedronate significantly increased the BMD change in Gruen zone 1. However, there was little clinical significance between the risedronate and control group in terms of the Gruen zones 2, 3, and 7. Oral risedronate significantly increased the Harris hip scores compared with the control group (*P* < 0.05).

**Conclusion:**

Oral risedronate could significantly reduce peri-prosthetic bone resorption around an uncemented femoral stem (Gruen zone 1) after THA. Due to the limited included studies, more high-quality randomized controlled trials (RCTs) were still needed to identify the efficacy of risedronate for bone loss in THA.

## Background

Total hip arthroplasty (THA) has become a popular and successful surgical option for patients with hip osteoarthritis or hip fracture [1]. Some studies revealed that more than 75% of the revision arthroplasties were performed due to prosthesis loosening and peri-prosthetic fracture, which were accompanied by severe periprosthetic bone loss [2]. If an ideal drug suppressing the bone resorption after THA was found, the service life of prosthesis would be much prolonged [3, 4].

Numerous studies have focused on peri-prosthetic bone metabolism after THA [5, 6]. Bone resorption is considered to be the main reason for prosthesis loosening [7]. Currently, bisphosphonates are anti-resorptive agents which promote bone mineralization and inhibit the biological effect of osteoclasts [8]. Many RCTs have demonstrated its beneficial effect on preserving periprosthetic bone in cementless THA [9, 10]. The risedronate has been used successfully to prevent osteoporotic fractures, mainly in the hip and vertebrae, by inhibiting osteoclast activity [11]. Risedronate can also reduce the risk of vertebral and hip fractures in patients with osteoporosis [12]. It could rapidly reduce bone turnover rates in adult patients at high risk of fractures [13]. In addition, risedronate has the potential efficacy in protecting against osteoporotic fractures and improving periprosthetic bone quality. Regardless of the potential efficacies of risedronate, no approved therapy for BMD loss associated with THA has been achieved due to the low evidence level of current articles.

Due to the potential positive effects, risedronate has been recommended to be used in THA as routine. However, there is less scientific evidence on the use of risedronate in preventing periprosthetic bone loss in THA. Thus, the purpose of this meta-analysis from RCTs was to evaluate whether oral risedronate could reduce femoral periprosthetic BMD loss and also increase the hip function in patients undergoing primary THA.

## Methods

This meta-analysis was reported according to the preferred reporting items for systematic reviews and meta-analyses (PRISMA) guidelines.

### Search strategy

We systematically searched papers in the following databases: PubMed, Embase, Web of Science, Cochrane Library, and Chinese Wanfang database. The following keywords were used in combination with Boolean operators AND or OR: “total hip replacement OR total hip arthroplasty” OR “THA” OR “THR” OR ““Arthroplasty, Replacement, Hip”[Mesh]” AND “risedronate.” No restrictions were imposed on language. The references of the relevant reviews were also reviewed to identify additional articles. All analyses were based on previous published studies; thus, no ethical approval and patient consent are required.

### Inclusion and exclusion criteria

#### Inclusion criteria

1. Participants: RCTs enrolling adult patients undergoing THA.
2. Interventions: Experimental groups received oral risedronate.
3. Comparisons: Control groups received equivalent placebo or no treatment.
4. Outcomes: Change in bone mineral density (BMD) in Gruen zones [14] and the Harris hip scores.
5. Study design: RCTs were considered as potentially relevant included articles in our study.

#### Exclusion criteria

Studies were excluded as follows: revision THA, patients suffer from an allergy to the risedronate, non-RCTs, letter, or without included outcomes.

### Selection criteria

Two reviewers independently reviewed the abstracts of the potential articles identified by the above searches. Subsequently, the full text of the studies that met the inclusion criteria were screened, and a final decision was made by discussion. A senior author had the final decision in any case of disagreement regarding which studies to be included.

### Data extraction

Two of the authors independently extracted data from the included studies. Corresponding authors were consulted for details of incomplete data. The following data were extracted and recorded in a spreadsheet: first author, publication year, sample size, baseline characteristics, intervention procedures, outcome, and duration of the follow-up. Other relevant data were also extracted from individual studies. Primary outcomes were changes in BMD in Gruen zones 1 and 7. Secondary outcomes were changes in BMD in Gruen zone 2, 3, 4, 5, and 6 and the Harris hip scores.

### Outcome measures and statistical analyses

The main outcomes were the changes in BMD in Gruen zones and the Harris hip scores. Continuous outcomes (Changes in BMD in Gruen zones and the Harris hip scores) were expressed as the weighted mean difference (WMD) and 95% confidence interval (CI). Statistical significance was set at *P* < 0.05 across the trials. Stata 12.0 (Stata Corp., College Station, TX) was used for the meta-analysis. Statistical heterogeneity was tested using *I*^2^ statistic. As the doses of risedronate were different, a random-effect model was chosen to avoid heterogeneity. Publication bias was not tested because the number of included studies was less than ten.

## Results

### Search results

**Fig. 1:**
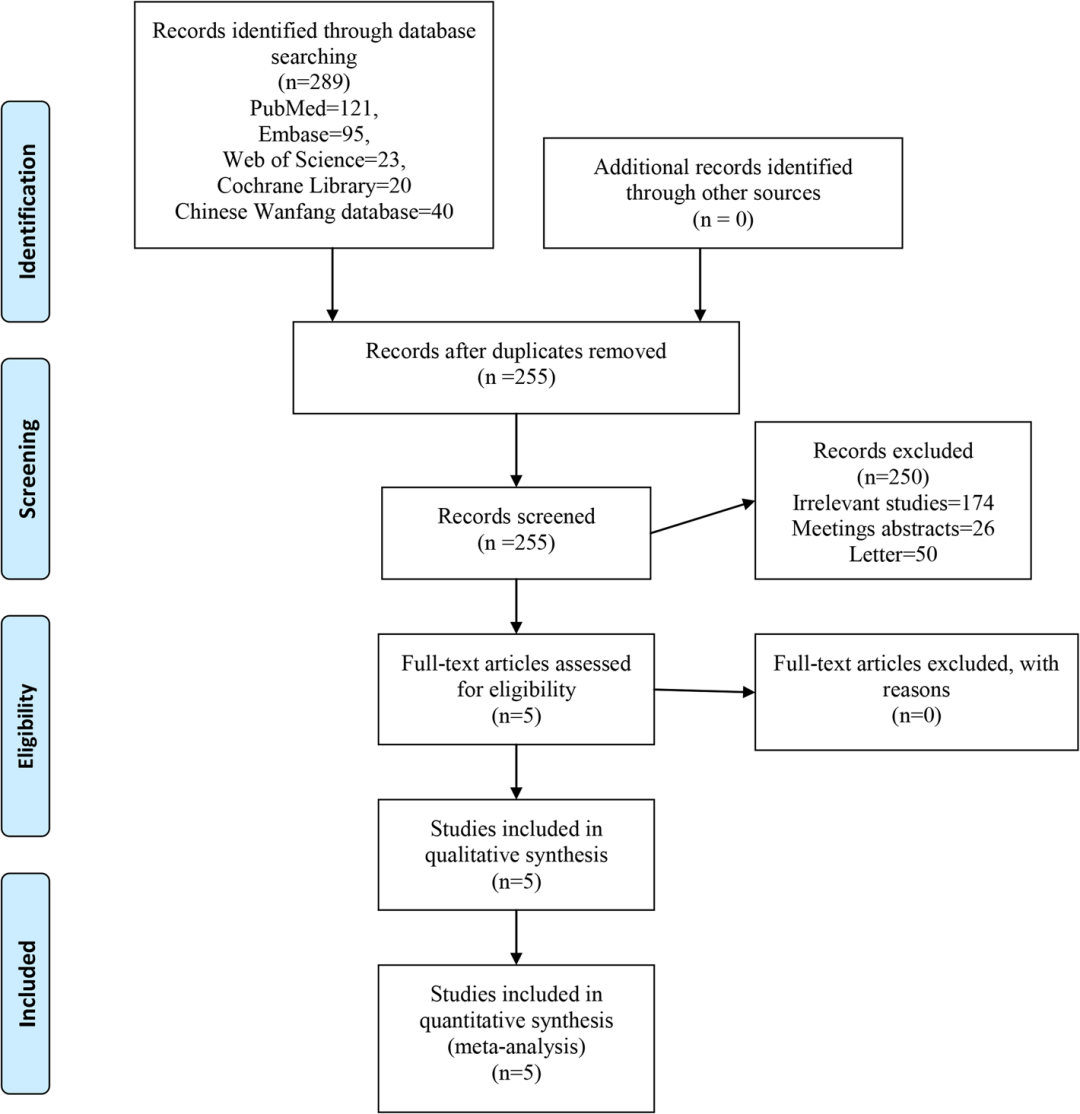
PRISMA flow chart of retrieved studies

The flow chart in Fig. 1 shows the screening process of the potential studies. A total of 289 studies (PubMed = 121, Embase = 95, Web of Science = 23, Cochrane Library = 20, Chinese Wanfang database = 40) were screened through the initial search. Two hundred fifty-five studies were then screened after duplicates were removed. Among these included studies, 280 studies were excluded on the basis of their titles and abstracts, and the remaining full text of 9 studies were read. After scanning the full text, four studies were also excluded since it did not meet inclusion criteria. Thus, five RCTs involving 259 patients (risedronate group = 127, control group = 132) were finally included in this meta-analysis [9–16].

### General characteristic and quality assessment

**Fig. 2:**
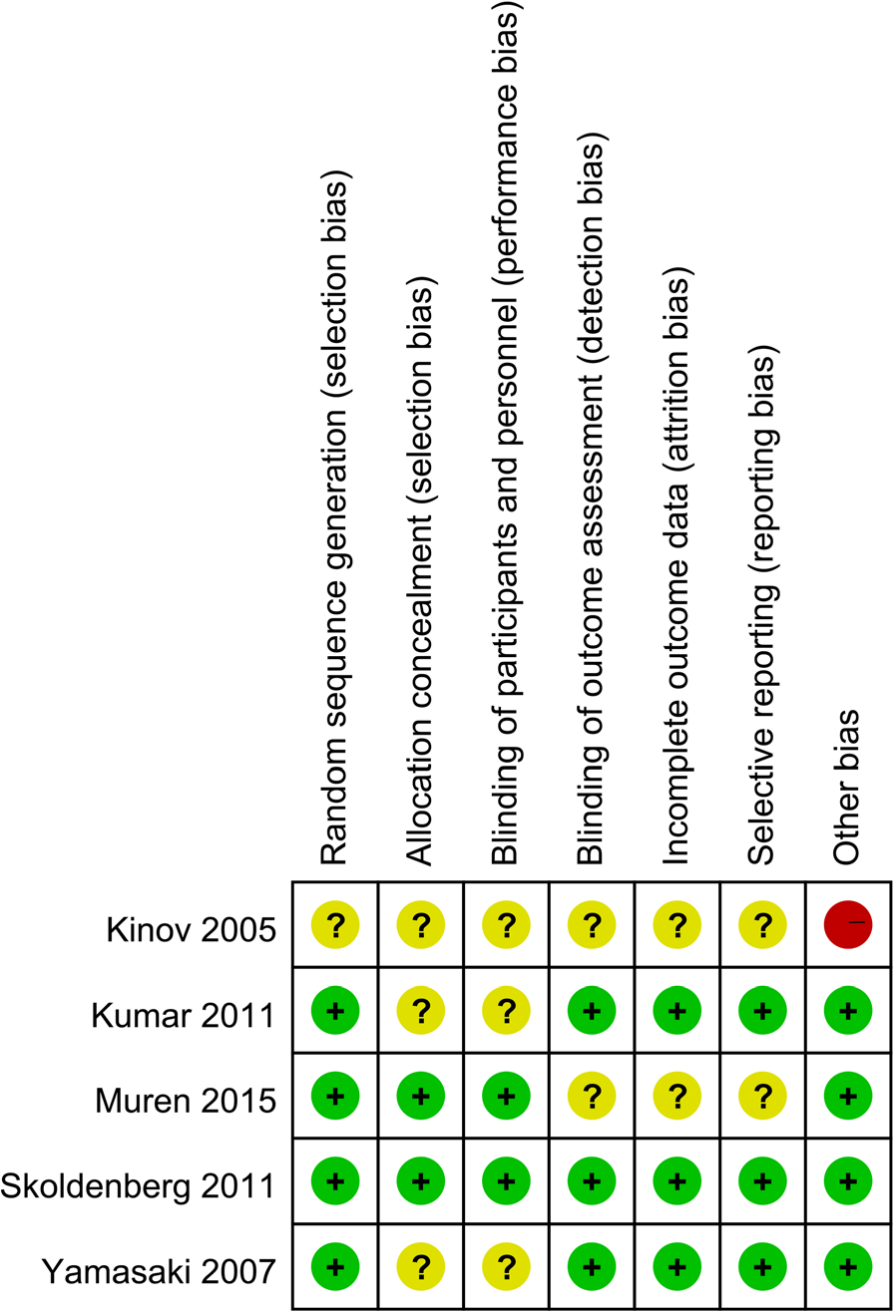
Risk of bias summary

**Fig. 3:**
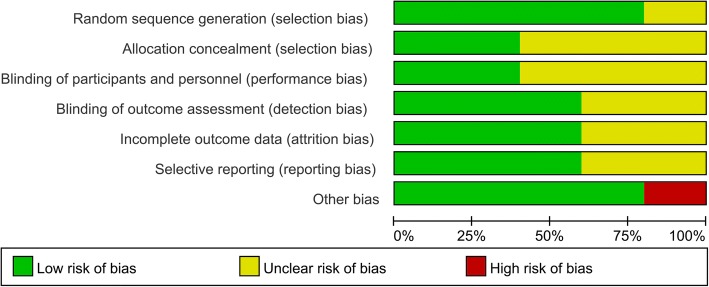
Risk of bias graph

Table 1 shows the basic characteristics of the included studies. All of the included studies were RCTs and published from the year of 2005. Four RCTs used 35 mg risedronate as intervention group and one RCT used 2.5 mg/day as intervention group. The duration of follow-up ranged from 6 months to 4 years. The quality assessment can be seen in Figs. 2 and 3. One study did not state the random sequence generation and listed as unclear risk of bias. Three studies did not state the allocation concealment and blinding of the participants and personnel and identified as unclear risk of bias. Two studies did not describe the blinding of outcome assessment and identify as unclear risk of bias.

**Table 1:** The general characteristic of the included studies

Author	Participant (E/C)	Mean age (year, E/C)	Male/female(%, E/C)	Intervention	Control	Outcomes	Follow-up	Study
Kinov 2005	12/12	NS	NS	35 mg risedronate	Placebo	1, 2, 8	6 months	RCTs
Yamasaki 2007	19/21	66.8/66.6	23.2/23.9	2.5 mg/day orally	Placebo	1, 2, 3, 4, 5	6 months	RCTs
Skoldenberg 2011	36/37	61/60	55/55	35 mg risedronate	Placebo	1, 2, 3, 4, 6, 7, 8	12 months	RCTs
Kumar 2011	30/31	60/61	59/61	35 mg risedronate	Placebo	1, 2, 3, 4, 5, 6, 7, 8	1 years	RCTs
Muren 2015	30/31	62/60	52/54	35 mg risedronate	Placebo	1, 2, 3, 4, 5, 6, 7, 8	4 years	RCTs

*E*, risedronate group; *C*, control group; *RCT*, randomized controlled trials; *1*, BMD change in zone 1; *2*, BMD change in zone 7; *3*, BMD change in zone 2; *4*, BMD change in zone 3; *5*, BMD change in zone 4; *6*, BMD change in zone 5; *7*, BMD change in zone 6; *8*, Harris hip scores

### Meta-analysis of BMD change in zone 1

**Fig. 4:**
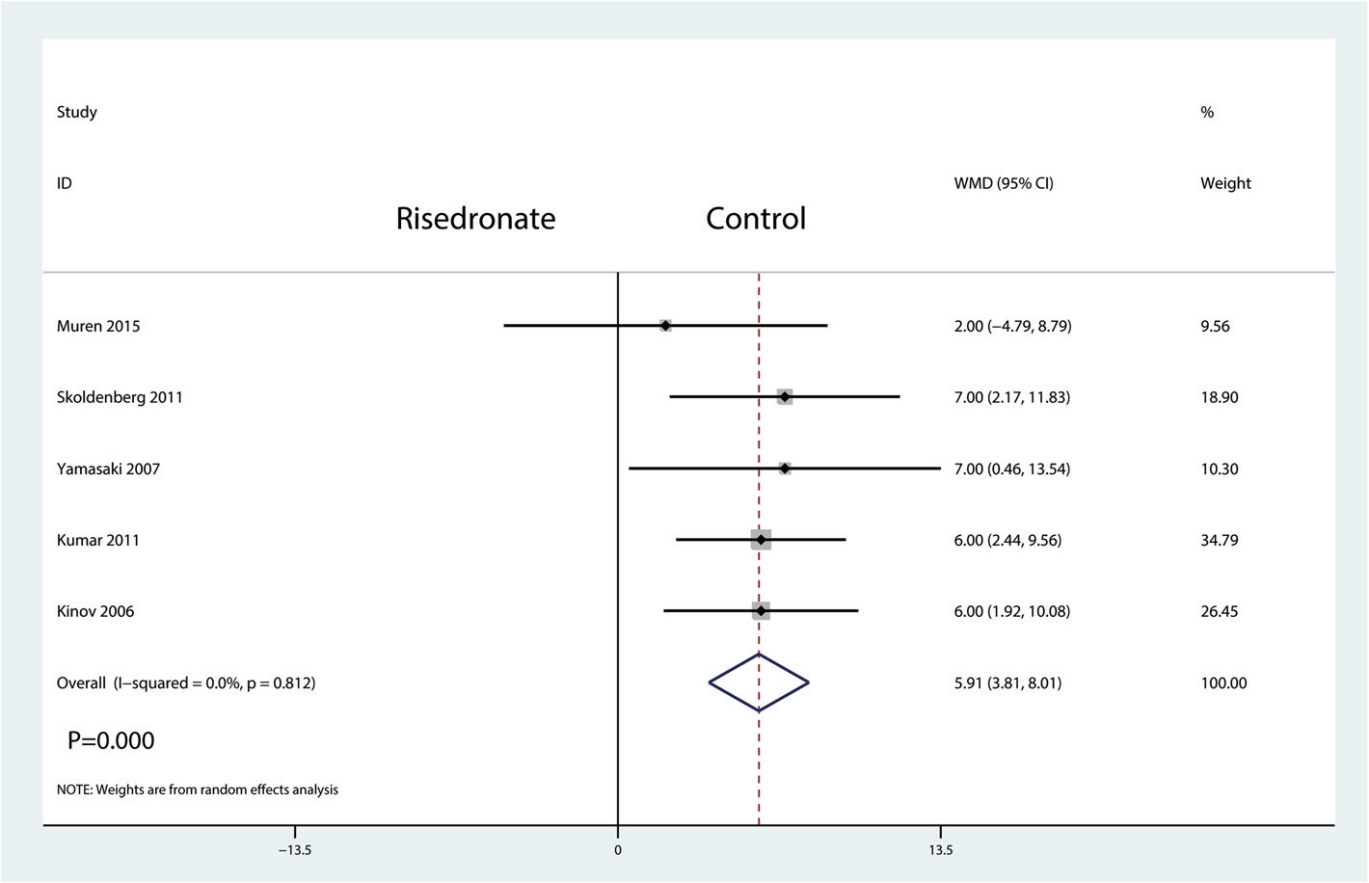
Forest plot comparing the BMD change in zone 1 between the two groups

A total of five studies involving 259 patients reported relevant data regarding BMD change in zone 1 (127 and 132 patients in the risedronate and control groups, respectively). The 259 patient outcomes from the meta-analysis indicated that oral risedronate significantly increased BMD change in zone 1 by a mean of 5.91 compared with the control group (WMD = 5.91, 95% CI 3.81 to 8.01; *P* = 0.000, *I*^2^ = 0.00%, Fig. 4).

### Meta-analysis of BMD change in zone 7

**Fig. 5:**
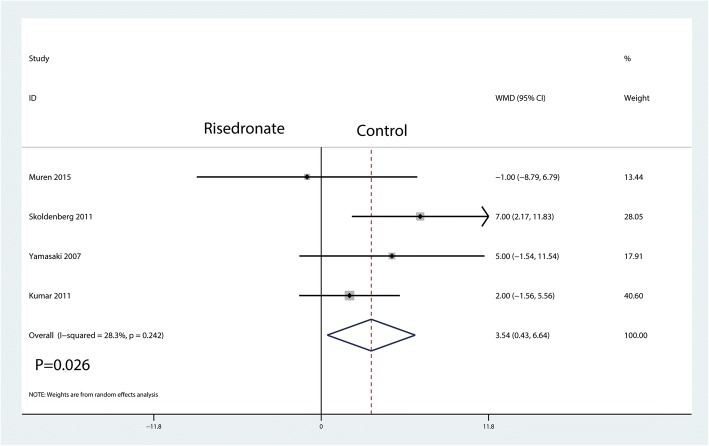
Forest plot comparing the BMD change in zone 7 between the two groups

A total of five studies involving 235 patients reported relevant data regarding BMD change in zone 7 (115 and 120 patients in the risedronate and control groups, respectively). The 235 patient outcomes from the meta-analysis indicated that oral risedronate significantly increased BMD change in zone 7 by a mean of 3.54 compared with the control group (WMD = 3.54, 95% CI 0.43 to 6.64; *P* = 0.026, *I*^2^ = 28.3%, Fig. 5).

### Meta-analysis of BMD change in zone 2

**Fig. 6:**
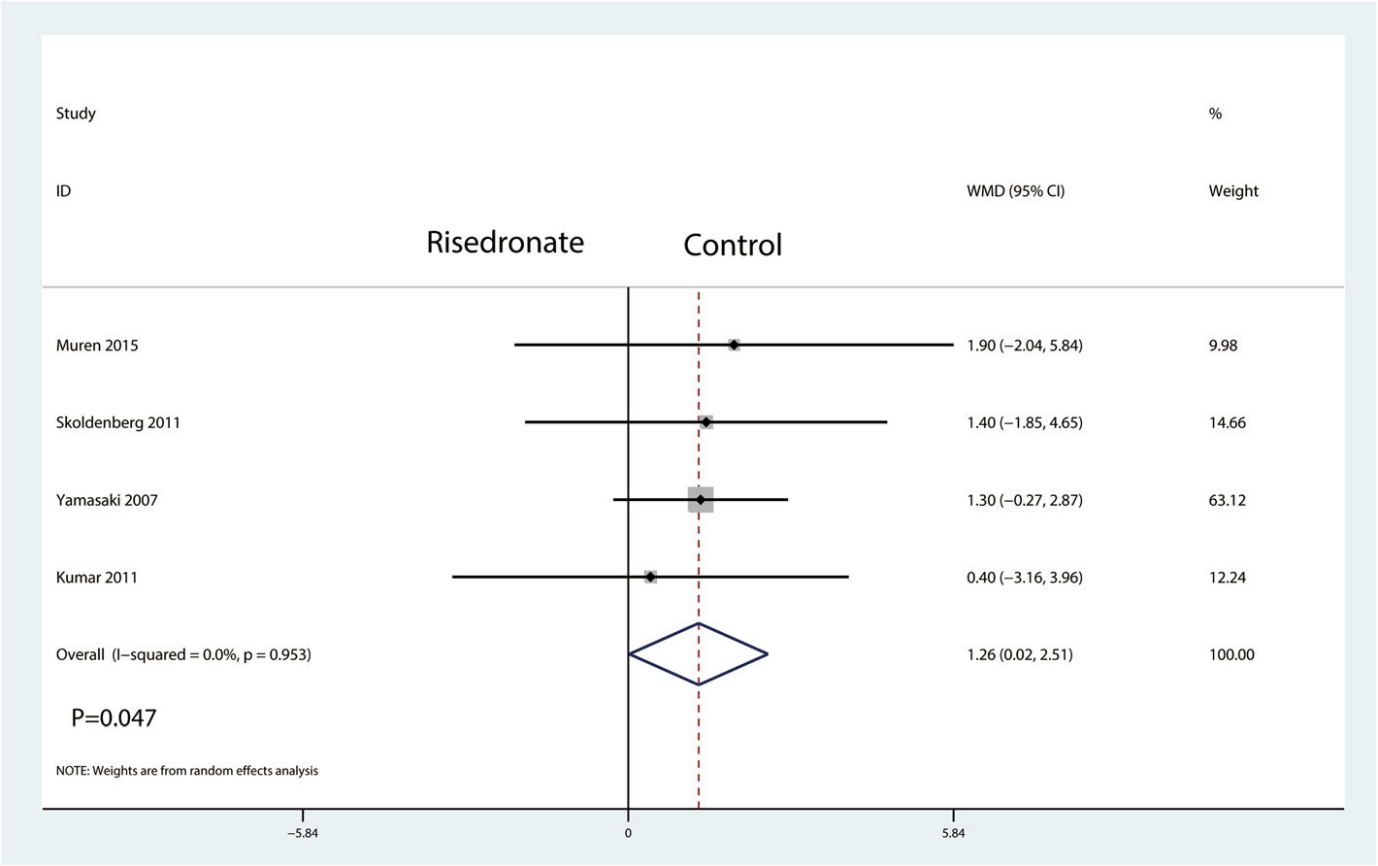
Forest plot comparing the BMD change in zone 2 between the two groups

A total of four studies reported relevant data regarding BMD change in zone 2 (115 and 120 patients in the risedronate and control groups, respectively). The 235 patient outcomes from the meta-analysis indicated that oral risedronate significantly increased BMD change in zone 2 by a mean of 1.26 compared with the control group (WMD = 1.26, 95% CI 0.02 to 2.51; *P* = 0.047, *I*^2^ = 0.0%, Fig. 6).

### Meta-analysis of BMD change in zone 3

**Fig. 7:**
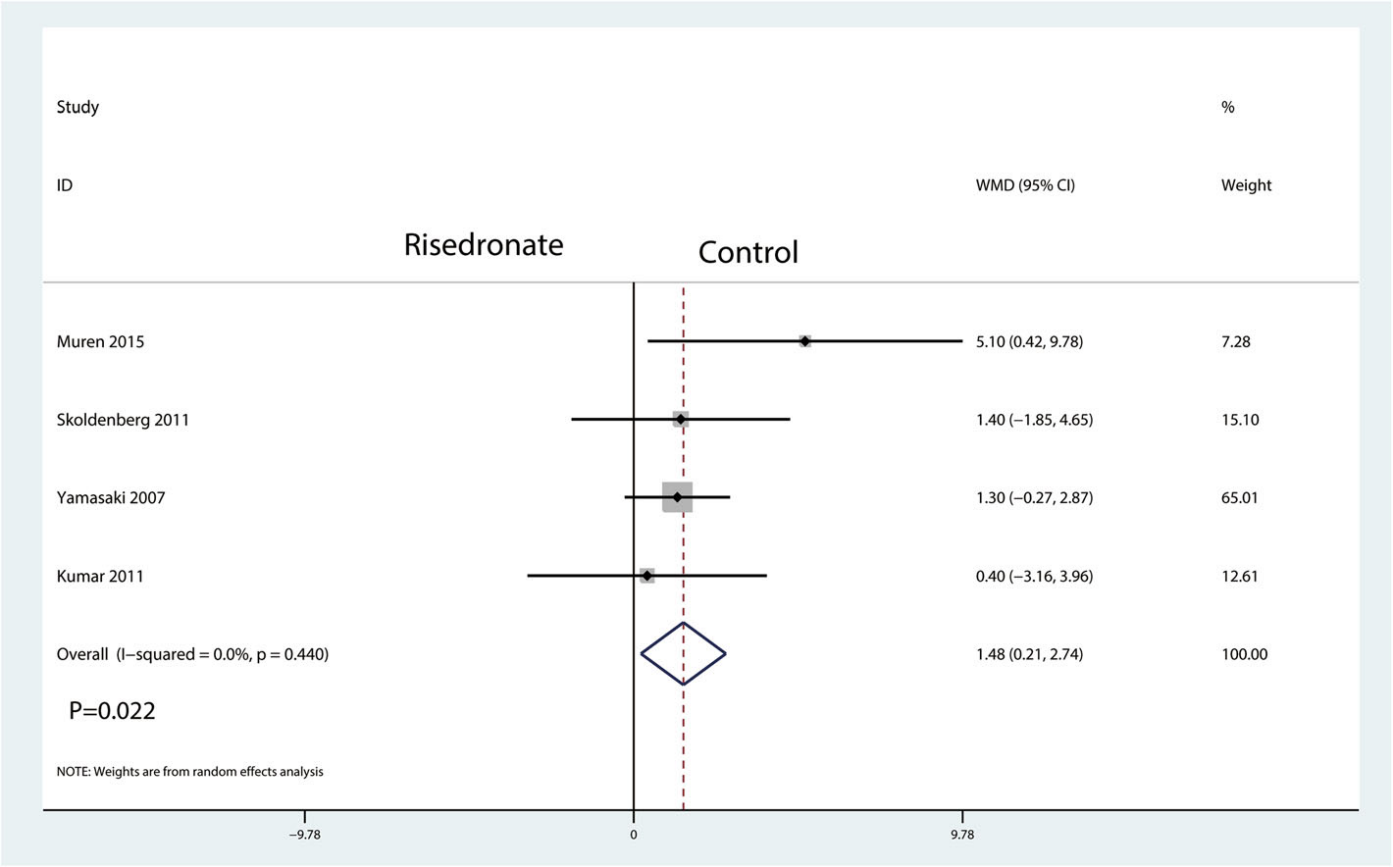
Forest plot comparing the BMD change in zone 3 between the two groups

A total of four studies reported relevant data regarding BMD change in zone 3 (115 and 120 patients in the risedronate and control groups, respectively). The 235 patient outcomes from the meta-analysis indicated that oral risedronate significantly increased BMD change in zone 3 by a mean of 1.48 compared with the control group (WMD = 1.48, 95% CI 0.21 to 2.74; *P* = 0.022, *I*^2^ = 0.0%, Fig. 7).

### Meta-analysis of BMD change in zone 4

**Fig. 8:**
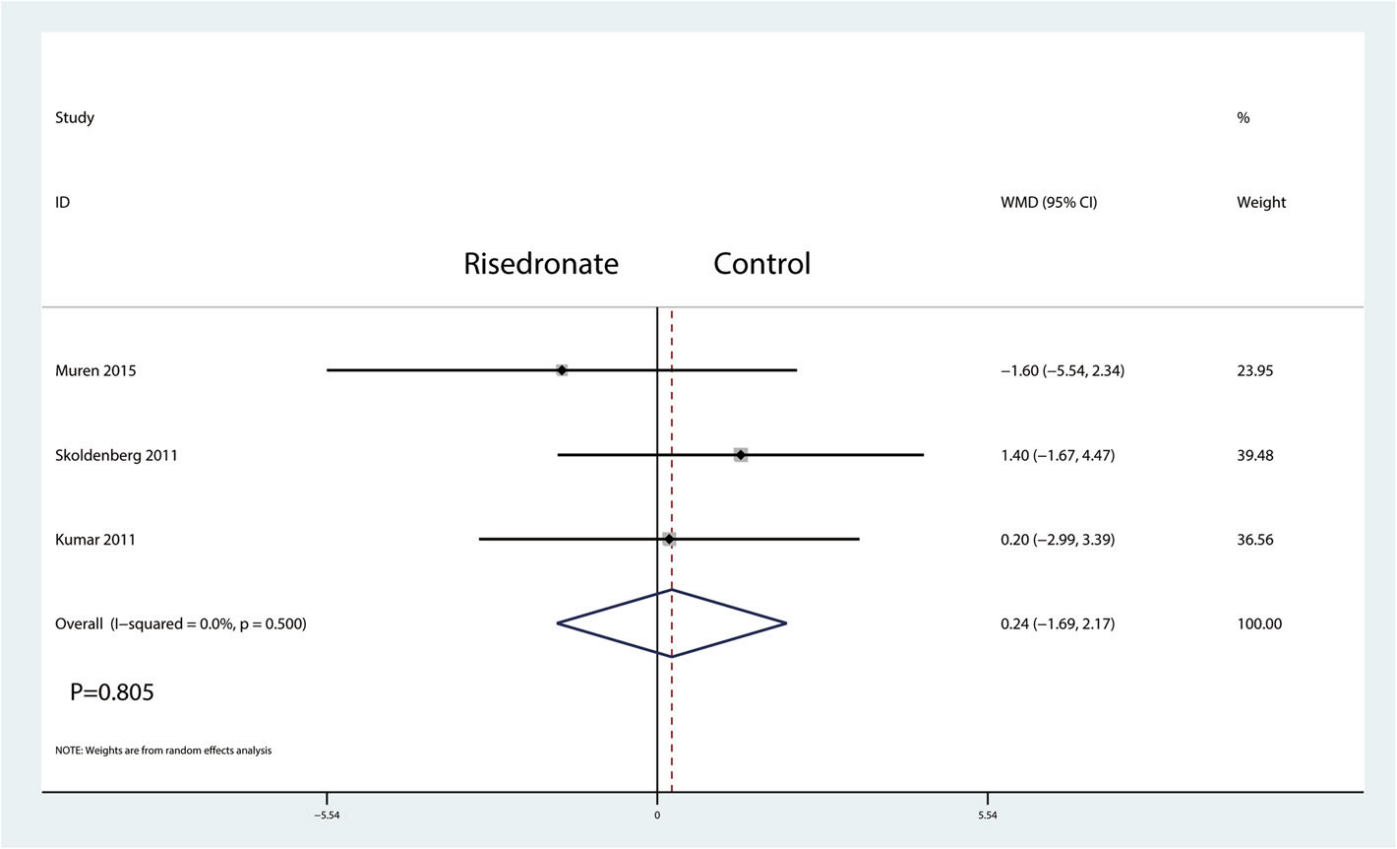
Forest plot comparing the BMD change in zone 4 between the two groups

A total of three studies reported relevant data regarding BMD change in zone 4 (96 and 99 patients in the risedronate and control groups, respectively). The 195 patient outcomes from the meta-analysis indicated that there was no significant difference between the BMD change in zone 4 (WMD = 0.24, 95% CI − 1.69 to 2.17; *P* = 0.805, *I*^2^ = 0.0%, Fig. 8).

### Meta-analysis of BMD change in zone 5

**Fig. 9:**
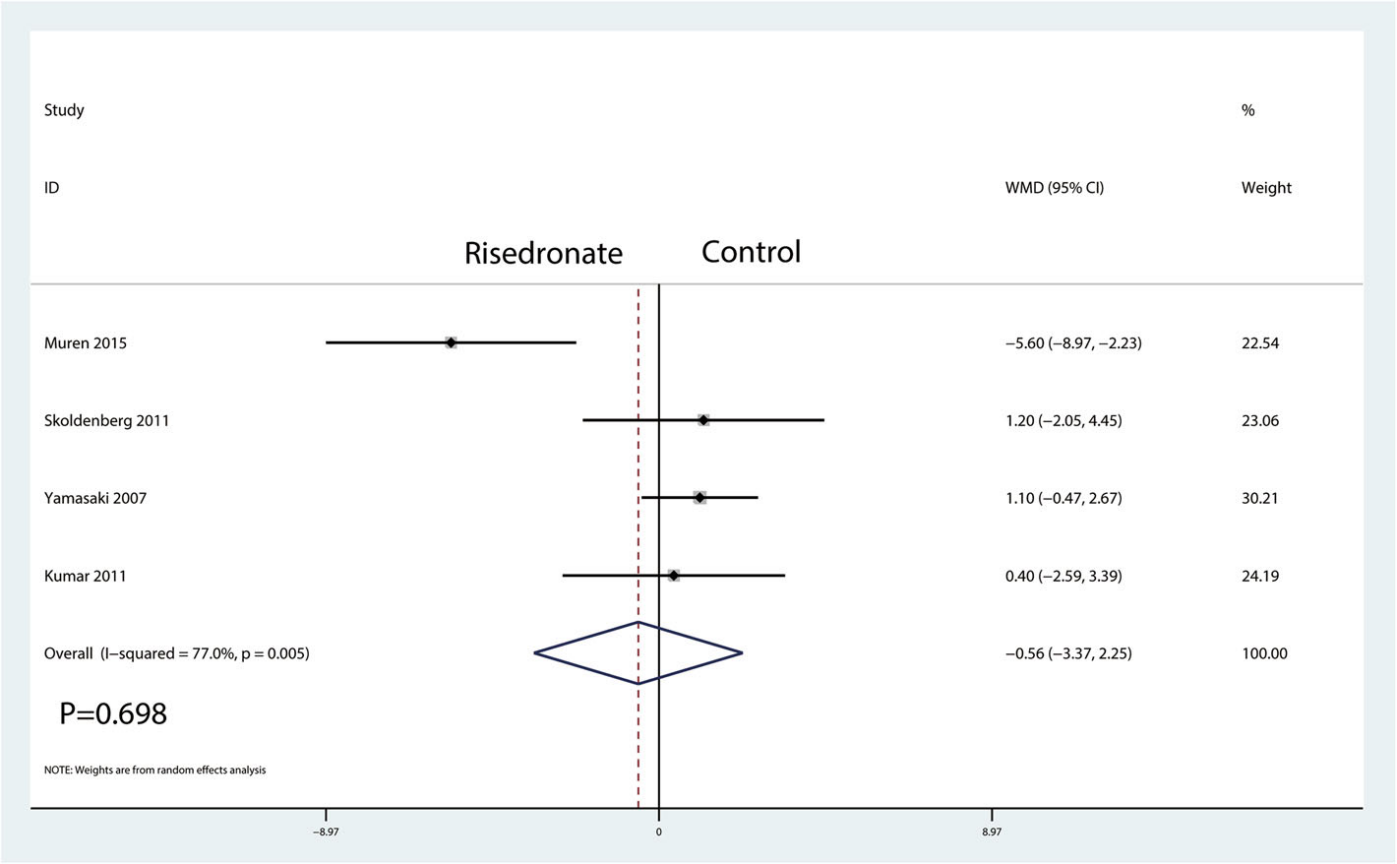
Forest plot comparing the BMD change in zone 5 between the two groups

A total of five studies reported relevant data regarding BMD change in zone 1 (115 and 120 patients in the risedronate and control groups, respectively). The 235 patient outcomes from the meta-analysis indicated that there was no significant difference between the risedronate and control groups regarding the BMD change in zone 5 (WMD = − 0.56, 95% CI − 3.37 to 2.25; *P* = 0.698, *I*^2^ = 77.0%, Fig. 9).

### Meta-analysis of BMD change in zone 6

**Fig. 10:**
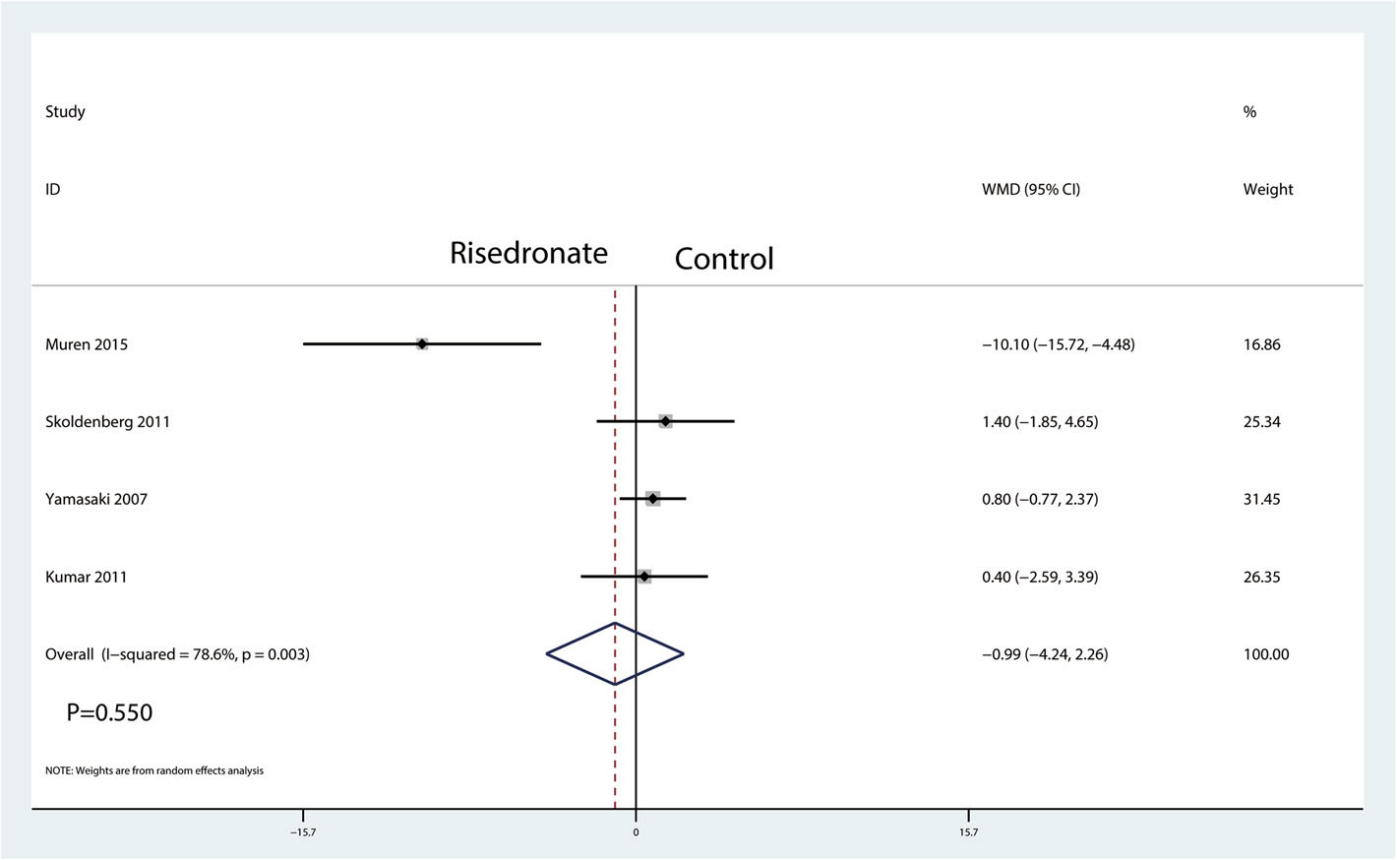
Forest plot comparing the BMD change in zone 6 between the two groups

A total of five studies reported relevant data regarding BMD change in zone 6 (326 and 325 patients in the risedronate and control groups, respectively). The 235 patient outcomes from the meta-analysis indicated that there was no significant difference between the risedronate and control groups regarding the BMD change in zone 5 (WMD = − 0.99, 95% CI − 4.24 to 2.26; *P* = 0.550, *I*^2^ = 78.6%, Fig. 10).

### Harris hip scores

**Fig. 11:**
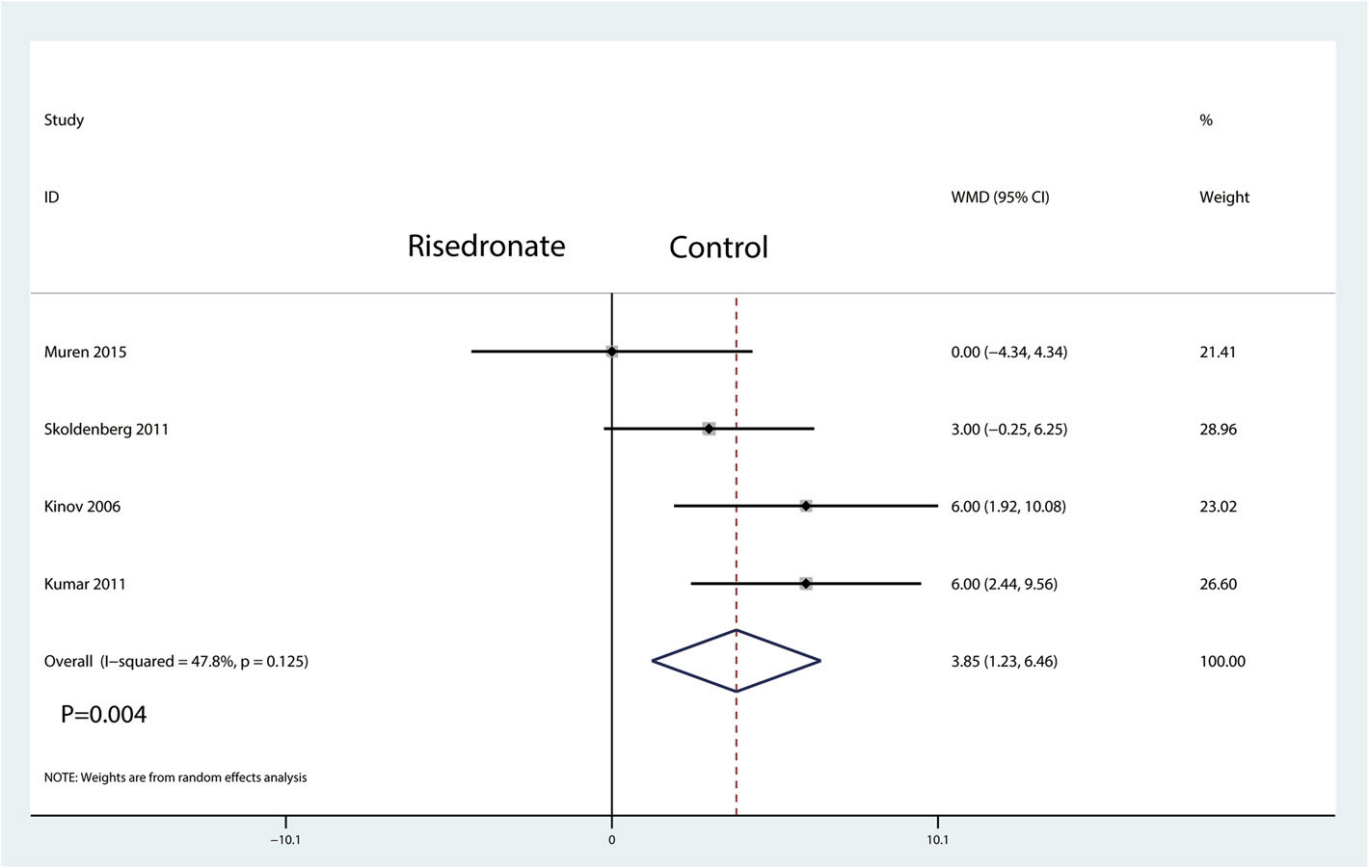
Forest plot comparing the Harris hip scores between the two groups

A total of four studies reported relevant data regarding Harris hip scores (108 and 111 patients in the risedronate and control groups, respectively). The 219 patient outcomes from the meta-analysis indicated that oral risedronate could increase the Harris hip scores compared with the control group (WMD = 3.85, 95% CI 1.23 to 6.46; *P* = 0.004, *I*^2^ = 47.8%, Fig. 11).

## Discussion

This is the first meta-analysis comparing the efficacy of oral risedronate in reducing bone loss after THA. Results indicated that oral risedronate can significantly increase the BMD around uncemented femoral stem (Gruen zone 1). There was little clinical significance between the risedronate and control groups in the Gruen zones 2, 3, and 7. Oral risedronate can also increase the Harris hip scores and thus has a positive role in improving the postoperative outcomes of the hip.

Our study was uniquely undertaken using the following approaches: (1) we systematically searched the electronic databases and calculated all of the outcomes with random-effect model, (2) our meta-analysis was performed and analyzed in accordance with the best practice methods recommended by the Cochrane Collaboration [17], and (3) only focused on the risedronate for bone loss after THA and thus avoid the clinical heterogeneity. In 2011, Prieto-Alhambra et al. [18] conducted a population-based parallel-cohort trial and indicated that bisphosphonates reduced the fracture risk among THA patients (hazard ratio (HR) = 0.56, 95% CI 0.38–0.82). We assessed the BMD around uncemented femoral stem (Gruen zones 1, 2, 3, 4, 5, 6, and 7). Final results indicated that oral risedronate had a positive role in increasing the BMD around uncemented femoral stem (Gruen zones 1, 2, 3, and 7). There was no significant difference between the BMD around uncemented femoral stem (Gruen zones 4, 5, and 6).

From a previous meta-analysis of 14 RCTs comparing bisphosphonates treatment with a placebo treatment in patients with total knee arthroplasty (TKA) and THA, Lin et al. [5] found that bisphosphonates reduced periprosthetic bone loss after TKA and THA. Although that finding was consistent with our research, that study was intended to investigate the efficacy and safety of bisphosphonates (alendronate, pamidronate, etidronate, zoledronate, risedronate, clodronate, and bisphosphonate) for patients with TKA and THA. Also, they only included one RCT that compared risedronate versus placebo for bone loss after THA and TKA. Thus, we could not conclude that risedronate had a significant influence on bone loss among patients with only THA. Zhao et al. [1] conducted a similar meta-analysis about bisphosphonates for bone loss after THA; however, only one study comparing risedronate versus placebo was included in the meta-analysis. Therefore, large-scale trials are required to validate the effects of risedronate focus on patients with THA.

The duration of the follow-up in the included studies was ranged from 6 months to 4 years. Obviously, the relative short use of bisphosphonate will decrease the efficacy of anti-resorption. Eberhardt et al. [19] reported that postoperative continuous and high-dose bisphosphonate treatment is potent in accelerating osseointegration of the prosthesis, which may prevent wear debris from migration by sealing the implant-bone interface. Friedl et al. [20] however doubt that the long-term efficacy of bisphosphonate could reduce bone loss after THA. We also found that oral risedronate could increase the Harris hip scores compared with the control groups.

The limitations of our study include the following: (1) the BMD results from the meta-analysis of some ROI appeared heterogeneous, and sensitivity analysis and subgroup analyses failed to eliminate the heterogeneity; (2) the included studies did not have sufficient duration of risedronate treatment and follow-up and also lacked evaluating indexes like functional scores and the rate of revision, so we could not evaluate the efficacy of postoperative risedronate treatment comprehensively; and (3) the number and quality of the included RCTs were limited and thus future high-quality RCTs were still needed to identify the efficacy of risedronate for bone loss after THA.

## Conclusions

In conclusion, oral risedronate could significantly reduce periprosthetic bone resorption around an uncemented femoral stem (Gruen zones 1, 2, 3, and 7) after THA. Due to the limited included studies and shortcoming of this meta-analysis, more high-quality RCTs are needed to identify the efficacy of risedronate for bone loss in THA.

## Abbreviations

BMD: Bone mineral density
CI: Confidence interval
HR: Hazard ratio
PRISMA: Preferred reporting items for systematic reviews and meta-analyses
RCT: Randomized controlled trials
THA: Tarthroplasty
WMD: Weighted mean difference
